# Marginal Structural Models to Assess Delays in Second-Line HIV Treatment Initiation in South Africa

**DOI:** 10.1371/journal.pone.0161469

**Published:** 2016-08-22

**Authors:** Julia K. Rohr, Prudence Ive, C. Robert Horsburgh, Rebecca Berhanu, Kate Shearer, Mhairi Maskew, Lawrence Long, Ian Sanne, Jean Bassett, Osman Ebrahim, Matthew P. Fox

**Affiliations:** 1 Center for Global Health & Development, Boston University, Boston, United States of America; 2 Clinical HIV Research Unit, Department of Internal Medicine, School of Clinical Medicine, Faculty of Health Sciences, University of the Witwatersrand, Johannesburg, South Africa; 3 Department of Epidemiology, Boston University School of Public Health, Boston, United States of America; 4 Health Economics and Epidemiology Research Office, Department of Internal Medicine, School of Clinical Medicine, Faculty of Health Sciences, University of the Witwatersrand, Johannesburg, South Africa; 5 Right to Care, Johannesburg, South Africa; 6 Witkoppen Health and Welfare Centre, Johannesburg, South Africa; 7 Department of Medical Microbiology, University of Pretoria, Pretoria, South Africa; Azienda Ospedaliera Universitaria di Perugia, ITALY

## Abstract

**Background:**

South African HIV treatment guidelines call for patients who fail first-line antiretroviral therapy (ART) to be switched to second-line ART, yet logistical issues, clinician decisions and patient preferences make delay in switching to second-line likely. We explore the impact of delaying second-line ART after first-line treatment failure on rates of death and virologic failure.

**Methods:**

We include patients with documented virologic failure on first-line ART from an observational cohort of 9 South African clinics. We explored predictors of delayed second-line switch and used marginal structural models to analyze rates of death following first-line failure by categorical time to switch to second-line. Cox proportional hazards models were used to examine virologic failure on second-line ART among patients who switched to second-line.

**Results:**

5895 patients failed first-line ART, and 63% switched to second-line. Among patients who switched, median time to switch was 3.4 months (IQR: 1.1–8.7 months). Longer time to switch was associated with higher CD4 counts, lower viral loads and more missed visits prior to first-line failure. Worse outcomes were associated with delay in second-line switch among patients with a peak CD4 count on first-line treatment ≤100 cells/mm^3^. Among these patients, marginal structural models showed increased risk of death (adjusted HR for switch in 6–12 months vs. 0–1.5 months = 1.47 (95% CI: 0.94–2.29), and Cox models showed increased rates of second-line virologic failure despite the presence of survivor bias (adjusted HR for switch in 3–6 months vs. 0–1.5 months = 2.13 (95% CI: 1.01–4.47)).

**Conclusions:**

Even small delays in switch to second-line ART were associated with increased death and second-line failure among patients with low CD4 counts on first-line. There is opportunity for healthcare providers to switch patients to second-line more quickly.

## Introduction

South Africa has the largest number of people living with HIV/AIDS worldwide, with roughly 5.9 million adults living with HIV in 2013 [1]. Now, over a decade after the launch of its national HIV program in 2004, many patients have failed first-line antiretroviral therapy (ART) and require second-line. In South Africa, approximately 14% of patients experience first-line virologic failure, and approximately 12% start second-line in 5 years after ART initiation [2]. Optimizing successful second-line treatment is essential, given limited options for third-line in South Africa and other resource-limited settings.

According to national treatment guidelines switch from first-line ART (2 nucleoside reverse transcriptase inhibitors (NRTIs) plus 1 non-nucleoside reverse transcriptase inhibitor (NNRTI)) to second-line (2 NRTIs plus 1 protease inhibitor (PI)) should occur immediately after two consecutive failing viral loads >1000 copies/mL on first-line, assuming a patient is adhering to treatment [3]. Patients with an elevated viral load are referred to adherence counseling and should have their viral load re-tested 3 months later. In practice, many patients switch late if at all [4], with second-line switch occurring in only about 62% of patients [5], and median time to switch occurring about 4.6 to 5 months after the second failing viral load measurement among those who switch [5,6]. Predictors of switching to second-line include low CD4 count at failure and a larger decline in CD4 count on first-line, indicating provider motivation to switch sicker patients more quickly [5,7]. On the other hand, clinicians can be reluctant to switch patients to second-line who are clinically well, due to cost and lack of possible future regimens [4,8].

Poor ART adherence has been consistently shown to predict treatment failure, viremia, and increase in drug resistance mutations [9–12]. Even low levels of resistance to first-line drugs have been associated with increased first-line virologic failure [13]. NRTI resistance is commonly found at first-line failure (as high as 81–90% of South African patients [14–20]), and an estimated 15–23% of South African patients failing first-line have resistance to NRTIs that could compromise second-line effectiveness [14,15]. Since protease inhibitor-based second-line regimens can re-suppress HIV even in the presence of drug resistance mutations, it is preferable to ensure failure is not due to adherence problems to avoid unnecessary switches [21,22]. Yet, leaving patients on treatment with long periods of viremia is harmful to patient health and increases NRTI resistance mutations, which could be detrimental to second-line regimen activity [4,16,23–29].

We investigate the impact of delaying second-line ART initiation on patient survival and second-line treatment outcomes. We also explore possible reasons for delays in second-line ART.

## Methods

### Data source and study population

We conducted an observational cohort study using medical record data from the Right to Care cohort, with patients from nine South African HIV clinics (seven in Gauteng, two in Mpumalanga Province). Each clinic used the same electronic medical record system, which captured patient demographics, clinical information, visit dates, lab results, diagnoses, and drug regimens. Retrospective analysis of the Right to Care cohort was approved by the Human Research Ethics Committee of the University of the Witwatersrand. Boston University provided permission for analysis of de-identified data.

The study population included treatment naïve adults (≥18 years old) who started a standard first-line ART regimen after April 2004 with confirmed first-line virologic failure. A standard regimen was defined as two NRTIs (stavudine (d4T), zidovudine (AZT) or tenofovir (TDF), plus lamivudine (3TC) or emtricitabine (FTC)), and one NNRTI (efavirenz (EFV) or nevirapine (NVP)). First-line failure was defined as two consecutive viral loads >1000 copies/mL between two weeks and 12 months apart, with the first failing viral load after at least three months on treatment. Patients who switched to second-line before documented treatment failure were excluded. Second-line was defined as switch from NNRTI to PI plus a change in at least one NRTI. The dataset closed in April 2014, so we only included subjects who experienced first-line failure before April 2013 to ensure that all patients had at least 12 months of potential follow-up after first-line failure to allow time for switch to second-line.

### Analysis

First, we used Cox models predicting days to switch after first-line failure to explore reasons for delayed switch to second-line. Relevant predictors were identified based on strength and precision of the hazard ratio. We considered variables related to clinic attendance (time between failing viral loads on first-line, missed visits on first-line) and disease progression (TB, CD4 count, viral load, and BMI at first-line failure), adjusting for clinic, year of failure, age and sex. We then categorized the population into mutually exclusive groups by time from first-line failure (date of second elevated viral load measurement) until second-line switch (<1.5 months, 1.5–3 months, 3–6 months, 6–12 months, >12 months, or never) to examine descriptive characteristics.

Next, we investigated the impact of delayed switch through two outcomes: (1) death after first-line failure, and (2) confirmed virologic failure on second-line (two consecutive viral loads >1000 copies/mL). All patients who failed first-line, including those who never switched, were included in analyses for death through the use of marginal structural models [30–34], while analyses for second-line failure were limited to patients who switched to second-line.

### Marginal structural models for death

In marginal structural models all patients who failed first-line contributed person-time to each time to switch exposure group until they failed to follow the definition of that exposure group, at which point they were censored. As a result, person-time and outcomes for patients who never switched were included, and time-dependent confounding was addressed. To create the marginal structural model, observations for each subject were created in half-month time units beginning at first-line ART failure. Each observation was matched to the most recent date of time-dependent covariates including BMI, hemoglobin, CD4 count and viral load, which were carried forward up to 12 months until the date of second-line switch. Person-time ended at death, dataset closure, or when an individual failed to follow routine viral load monitoring, defined as >12 months without a viral load measurement. The dataset was then expanded to make 6 copies of each individual’s follow-up time, one for each exposure group: switch in <1.5 months, 1.5–3 months, 3–6 months, 6–12 months, >12 months, or never (illustrated in S1 Fig).

Artificial censoring in these models created selection bias that was accounted for by weighting by the inverse probability of remaining uncensored. Probability of remaining uncensored is equivalent to the probability of starting (or not starting) second-line at a given time point for each exposure group [30]. Probabilities of second-line initiation were calculated in the unexpanded data, using person-time until the half-month of second-line treatment initiation, if it occurred. Stabilized weights were calculated for each person-half-month using two pooled logistic regression models to calculate the probability of initiating second-line. The numerator model estimated the probability a subject had their observed exposure given baseline confounders (time, time^2^, clinic, year of failure, gender, age, viral load and CD4 at first-line failure) and exposure history. The denominator model estimated the probability a subject had their observed exposure given baseline (as above), time varying confounder values (CD4 count and BMI) and exposure history. The resulting weights had a mean of 1.02 and a range from 0.30 to 4.19.

Marginal structural Cox model parameters were estimated by fitting a weighted pooled logistic regression to estimate hazard of death by time of switch to second-line group. Robust standard errors were used to account for weighting. We adjusted for variables included in the numerator of the weight model and considered other confounders that were common causes of time to switch and death. Models were stratified by several potential effect measure modifiers related to patient health on first-line, including peak CD4 count from ART initiation through first-line failure, viral load at first-line failure, and viral suppression on first-line ART.

### Models for second-line failure

For analyses with second-line virologic failure as the outcome, we examined categorical time to switch to second-line (switch in <1.5 months, 1.5–3 months, 3–6 months, 6–12 months, >12 months) using Cox proportional hazards models. Individuals who never switched were not included. Person-time began at time of second-line switch, and ended at the outcome of interest or when a patient failed to follow routine virologic monitoring on second-line. Outcomes for a single elevated viral load and virologic failure after 3 months on second-line were included in sensitivity analyses. Inverse probability of censoring weights were used in models for both death and second-line failure to explore the impact of patients lost to follow-up after switch to second-line [35].

## Results

### Study population

We included 5895 patients who failed first-line ART. 370 patients were excluded because they switched to second-line before failure, and 991 patients were excluded because they failed treatment <12 months before the dataset closure. Median time between failing viral loads on first-line was 2.9 months (IQR: 1.9–4.9). Median time on first-line until failure was 17.4 months (IQR: 10.2–30.9). There were 2189 patients (37%) who never switched to second-line, and only 28% of these had evidence of a suppressed viral load within 12 months after failure. Among the 3706 who switched to second-line, median time to switch after first-line failure was 3.4 months (IQR: 1.1–8.7 months). Median follow-up on second-line among patients who switched was 16.8 months (IQR: 8.5–28.9 months).

After switch to second-line, 14% experienced confirmed virologic failure on second-line, 42% remained in care with regular viral load monitoring, 12% remained in care but had missed viral load monitoring visits, 30% stopped attending the clinic (had their last clinic attendance date >6 months prior to the dataset closure), and 2% died. Among those who stopped attending the clinic, 42% were recorded as transfers, and 8% later died. Table 1 describes the population at first-line failure by time to switch group.

**Table 1:** Description of study population at time of first-line failure, split by time to switch to second-line group.

	0 to 1.5 mo.	1.5 to 3 mo.	3 to 6 mo.	6 to 12 mo.	>12 mo.	Never
N (%)	1114	669	688	593	642	2189
	(18.9%)	(11.4%)	(11.7%)	(10.1%)	(10.9%)	(37.1%)
Gender (% female)	64.8%	60.8%	60.6%	61.9%	64.6%	63.1%
Age (median, IQR)	37.2	37	37	37.1	36.4	36.9
	(32.0–44.1)	(31.8–43.7)	(32.1–43.5)	(32.0–43.5)	(30.7–42.8)	(31.6–43.1)
Viral load (copies/mL; median, IQR)	19000	15862	12741	14000	12000	9400
	(5000–80000)	(4500–66000)	(4100–53000)	(4034–55649)	(3381–52355)	(2720–58344)
CD4 (cells/mm^3^; median, IQR)	185	191	197	182.5	207	221
	(103–299)	(107–312)	(108–287)	(100–286.5)	(122–326)	(118–346)
BMI (median, IQR)	24.2	24	23.8	24.3	24.3	23.8
	(21.2–27.9)	(21.5–27.9)	(21.1–27.6)	(21.5–28.2)	(21.4–27.9)	(20.9–27.6)
Hemoglobin (g/dL; median, IQR)	13.1	13	13	13	12.9	13
	(11.7–14.2)	(11.8–14.2)	(11.8–14.2)	(11.7–14.2)	(11.6–14.4)	(11.6–14.1)
Median percent of missed visits before failure	6.1%	6.7%	7.7%	8.4%	9.1%	6.9%
Percent with no missed visits in first 6 months after failure	69.7%	68.4%	61.6%	56.8%	56.7%	58.2%

### Predictors of time to switch

Patients who did not switch or switched in >12 months tended to have lower viral loads and higher CD4 counts at first-line failure than those who switched more quickly (Table 1), suggesting clinicians may delay switch to optimize adherence and preserve first-line ART. Similarly, in Cox models, low CD4 count and high failing viral load at first-line failure predicted quicker switch to second-line. The adjusted hazard ratio ([aHR], in a model with clinic, year of failure, time between first-line failing viral loads, age, sex, CD4 count and viral load at first-line failure) for switch to second-line among patients with a failing viral load >60,000 copies/mL compared to 1,000–9,999 copies/mL was 1.34 (95% CI: 1.21–1.49). The aHR for patients with CD4 count at first-line failure of 0–49 vs. >250 cells/mm^3^ was 1.32 (95% CI: 1.14–1.52).

Delayed switch was also related to patient behavior. Missing visits on first-line before failure was more common for patients with longer time to switch (Table 1). In Cox models the aHR for switch among those missing <5% of visits prior to first-line failure vs. missing >20% was 1.20 (95% CI: 1.08–1.35).

### Marginal structural models for death

Marginal structural models for death after first-line failure included all patients who failed first-line, even if they did not switch to second-line. There were 298 (5.1%) patients who had person-time end at time of death. Remaining patients were lost to follow-up (3555 (60.3%)), or remained in care (2042 (34.6%)). For 152 patients, death occurred after loss to follow-up, and they were considered lost and not a death. Models were adjusted for proportion of missed visits prior to first-line failure, which is predictive of time to switch and death, and all variables included in the numerator of the weights other than clinic due to small strata. We found that longer time to switch groups had slightly elevated hazards of death (aHR for switch >12 vs. 0–1.5 months: 1.21; 95% CI: 0.95–1.54) (Table 2). After stratifying by peak CD4 count prior to first-line failure, the harmful effect of delaying switch to second-line was more pronounced among the sickest patients with a peak CD4 count ≤100 cells/mm^3^ (aHR switch in >12 vs. 0–1.5 months: 1.53; 95% CI: 0.99–2.37). Among patients with a peak CD4 count >100 cells/mm^3^, delayed switch to second-line was associated with slightly elevated death rates only among the longest time and never switch groups, while patients switching <6 months after first-line failure had similar hazards of death as patients who switched within 1.5 months (Table 2).

**Table 2:** Adjusted marginal structural models for hazard ratios of death after first-line failure, stratified by peak CD4 count prior to first-line failure.

		All patients	Peak CD4 ≤ 100 cells/mm^3^ prior to first-line failure	Peak CD4 > 100 cells/mm^3^ prior to first-line failure
		(N = 3706)	(N = 496)	(N = 2939)
		aHR* (95% CI)	aHR (95% CI)	aHR (95% CI)
Time to switch:	0–1.5 months	Ref	Ref	Ref
	1.5–3 months	1.13 (0.88, 1.46)	1.34 (0.86, 2.09)	1.02 (0.75, 1.38)
	3–6 months	1.16 (0.90, 1.48)	1.37 (0.88, 2.12)	1.06 (0.78, 1.43)
	6–12 months	1.20 (0.94, 1.53)	1.47 (0.94, 2.29)	1.12 (0.84, 1.50)
	>12 months	1.21 (0.95, 1.54)	1.53 (0.99, 2.37)	1.11 (0.83, 1.48)
	Never	1.26 (0.99, 1.60)	1.54 (0.99, 2.38)	1.16 (0.87, 1.55)

*Adjusted for year of failure, sex, age, viral load at first-line failure, CD4 count at first-line failure, missed visits prior to first-line failure. Stratum for peak CD4 count ≤ 100 cells/mm3 prior to first-line failure was not adjusted for CD4 count at first-line failure due to small strata.

Marginal structural models stratified by other indicators of patient health on first-line (viral load at first-line failure, viral suppression on first-line ART) similarly demonstrated that delaying switch was most harmful for sickest patients (S1 Table). When excluding patients who had ≥8 months between first-line failing viral loads, the association between delayed switch and death became stronger, showing a 74% increase in hazards of death among the sickest patients for those switching in >12 months compared to those switching in 0–1.5 months (S2 Table).

### Models for second-line failure

When examining second-line treatment failure, individuals who did not switch could not be included. As a result, there was survivor bias in the Cox models for second-line treatment failure because patients in longer time to switch groups survived longer after first-line failure by definition. A substantial proportion of non-switching patients were lost from care soon after first-line failure, and the loss of sicker patients more quickly after first-line failure emphasizes the likely presence of survivor bias. Among patients who never switched, 34% (100/294) of those with a low peak CD4 count (≤100 cells/mm^3^) did not return to the clinic after 3 months following first-line failure, compared to 17% (319/1877) with a peak CD4 count >100 cells/mm^3^.

Unlike models for death where longer time to switch groups tended to have worse outcomes, the longest time to switch groups were associated with reduced rates of failure on second-line ART, likely due to survivor bias. Like the models for death, the second-line treatment failure results differed when stratified by peak CD4 count prior to first-line failure. Among very sick patients with peak CD4 counts ≤100 cells/mm^3^ prior to first-line failure, even a small delay in switch was associated with worse second-line outcomes despite survivor bias, but the harmful effect of delay was attenuated as time to switch group became longer than six months (Table 3).

**Table 3:** Adjusted Cox proportional hazards ratios for confirmed failure on second-line ART stratified by peak CD4 count on treatment prior to first-line failure.

	Peak CD4 ≤ 100 cells/mm^3^ prior to first-line failure (N = 496)		Peak CD4 > 100 cells/mm^3^ prior to first-line failure (N = 2939)	
Months to switch	N	aHR*	N	aHR*
0 to 1.5	156	Ref	884	Ref
1.5 to 3	99	2.26 (1.12, 4.55)	524	0.87 (0.67, 1.14)
3 to 6	101	2.13 (1.01, 4.47)	552	1.03 (0.78, 1.35)
6 to 12	87	1.54 (0.68, 3.46)	460	0.87 (0.63, 1.20)
>12	53	1.35 (0.49, 3.73)	519	0.73 (0.52, 1.02)

*Adjusted for gender, age, viral load level at failure, BMI at failure, year of failure, missed visits before failure, time on first-line ART, clinic.

Death as a competing risk was evaluated using graphical methods [36] and impacted the second-line failure rate by <1% over 3 years, so deaths prior to failure were censored at time of death. Alternative second-line virologic outcomes (a single elevated viral load, virologic failure after 3 months on second-line) also showed worse outcomes associated with delayed switch for patients ≤ 100 cells/mm^3^ prior to first-line failure (S3 Table). Both Cox models for second-line failure and marginal structural models for death using inverse probability of censoring weighting to correct for loss to follow-up showed consistent findings (S4 Table).

## Discussion

Delaying second-line was associated with an increased death rate, especially among sickest patients. Among patients with peak CD4 counts ≤100 cells/mm^3^ prior to first-line failure, delaying second-line increased the death rate after first-line failure by close to 40% for a 6-month delay, and >50% for a delay over 6 months (Table 2). Among relatively healthier patients at first-line failure, delaying switching by up to 6 months did not influence the death rate, which highlights the efficacy of second-line ART for the majority of patients. Our finding was consistent with previous evidence that remaining on first-line after virologic failure leads to increased mortality compared to patients who switch to second-line [37–39]. Our study additionally provides specific estimates for relative hazards of death based on the time category of delay in switch, and demonstrates that the harmful effect of delayed switch differs by CD4 count status on first-line, although confidence intervals were fairly large due to the smaller stratified sample.

Analyzing predictors of time to switch provided insight into who switched to second-line most quickly. Unlike a previous study in South Africa that found a slight reduction in switch to second-line among patients with highest viral loads, likely due to provider hesitancy to switch patients they believe are non-adherent [7], we found patients with the highest viral loads and lowest CD4 counts switched most quickly. The majority of patients, even those who never switched, missed no visits during the 6 months following first-line failure, indicating that delay in switch may be partly due to provider factors and that there is opportunity to switch patients more quickly.

Several biases could impact this study. First, residual confounding due to poor adherence and residual confounding by indication are likely present despite adjustment for variables related to patient health and adherence prior to first-line failure. Confounding by indication due to sicker patients being switched more quickly would bias results showing a harmful effect of delayed switch toward the null. Second, differential surveillance could be a concern if patients who were switched most quickly had failure on second-line identified most quickly because of increased treatment monitoring following switch to second-line ART. However, median time to next viral load following switch was consistent across groups, and differential surveillance would not affect models for death. Third, survivor bias was present in Cox models for outcomes on second-line ART, yet despite this bias we saw evidence of increased second-line treatment failure with longer time to switch among the sickest patients. Models for death following first-line treatment avoided this bias by including patients who never switched. Lastly, selection bias may have occurred due to differential loss to follow-up after second-line switch. Our models using inverse probability of censoring weighting to adjust for loss to follow-up showed similar results. Still, the high rate of loss to follow-up represents a public health threat of transmitted drug resistance, and highlights a significant challenge in reaching the UNAIDS 90-90-90 target to keep 90% of diagnosed patients on sustained ART [40].

### Possible mechanisms

The association between delay in second-line initiation and increased death or virologic failure on second-line may be explained by clinical factors such as accumulation of drug resistance mutations among the sickest patients, and decline in immune system function and overall health in the setting of ongoing viremia. Poor adherence on first-line is an important predictor of poor adherence on second-line, which would cause negative treatment outcomes [9–12,41], but we attempted to control for adherence prior to first-line failure to focus on mechanisms caused by delayed second-line treatment.

NRTI resistance mutations that can compromise second-line ART accumulate as patients remain on failing regimens [16,22,25,26,28,29,42]. Thymidine analogue mutations (TAMs) accumulate rapidly while patients are on a failing NNRTI-based regimen [4,22,42], and prolonged time on failing regimens has been associated with the presence of ≥3 TAMs [28], which would potentially limit the effectiveness of NRTIs on second-line. Emergence of TDF-associated resistance mutations has been observed most often among patients with CD4 counts <100 cells/mm^3^ [25]. Delaying switching would allow more NRTI resistance mutations to develop, potentially to a greater extent in patients with CD4 <100 cells/mm^3^. Although suppression in the near-term on a second-line regimen is possible with NRTI resistance mutations [17,22,43], questions remain about the durability of second-line ART in the presence of NRTI resistance [25,26].

Prolonged viremia leading to clinical progression of HIV disease can also account for increased mortality rates and treatment failure in those with delayed switch to second-line, irrespective of drug resistance mutations. Exposure to viremia for a longer period is associated with higher mortality [23,24], and second-line treatment in Africa is less effective with high baseline viral load, likely a sign of prolonged viremia on first-line [44]. AIDS defining events prior to second-line switch are also associated with worse second-line outcomes [45].

### Future direction

We saw that even short delays in second-line ART initiation can be clinically harmful, particularly for the sickest patients, and that healthcare workers often have an opportunity to switch patients to second-line earlier. While logistical barriers to rapid switch may remain, there is a need for greater provider education on the importance of rapid switch to second-line after first-line failure particularly among these patients. In future research, further investigation into the reasons for delayed switch at the provider level, as well as drug resistance data, would help elucidate pathways between delayed switch, death, and second-line treatment failure.

## Supporting Information

S1 Fig Illustration of allocation of person time in marginal structural models. Hypothetical person time contributed to each of the 6 exposure groups in marginal structural models.
(DOCX)

S1 Table Alternative stratifications for adjusted marginal structural models for hazard ratios of death after first-line failure. (DOCX)

S2 Table Adjusted marginal structural model hazard ratios for death after first-line failure, limiting to patients with 2 weeks to <8 months between failing viral loads on first-line (n = 4908). (DOCX)

S3 Table Adjusted Cox proportional hazards ratios for alternative virologic outcomes on second-line ART, stratified by peak CD4 count prior to first-line failure. (DOCX)

S4 Table Adjusted marginal structural models for hazard ratios of death after first-line failure (a) and adjusted Cox proportional hazards ratios for confirmed failure on second-line ART (b), with weighting by inverse probability of censoring after second-line switch to account for loss to follow-up.
(DOCX)
