# Supplementary material for: Marginal Structural Models to Assess Delays in Second-Line HIV Treatment Initiation in South Africa
Source: PLoS One. 2016 Aug 22;11(8):e0161469. doi: 10.1371/journal.pone.0161469 (PMC4993510; doi:10.1371/journal.pone.0161469)

**SUPPLEMENT**

**S1 Figure. Illustration of allocation of person time in marginal structural models.**

Hypothetical person time contributed to each of the 6 exposure groups in marginal structural models, for an individual who switches to second-line ART in 1 month (person 1), never (person 2), in 8 months (person 3), and in 4 months (person 4). Time of switch to second-line is represented by a red-line. For example, person 1 switches in 1 month, and contributes all of their person time to exposure group “switch in 0 – 1.5 months”. At 1 month they fail to follow the definition of the other exposure groups and their follow-up time is censored. For each individual, person-time was censored for each exposure group based on the time at which an individual switched to second-line.


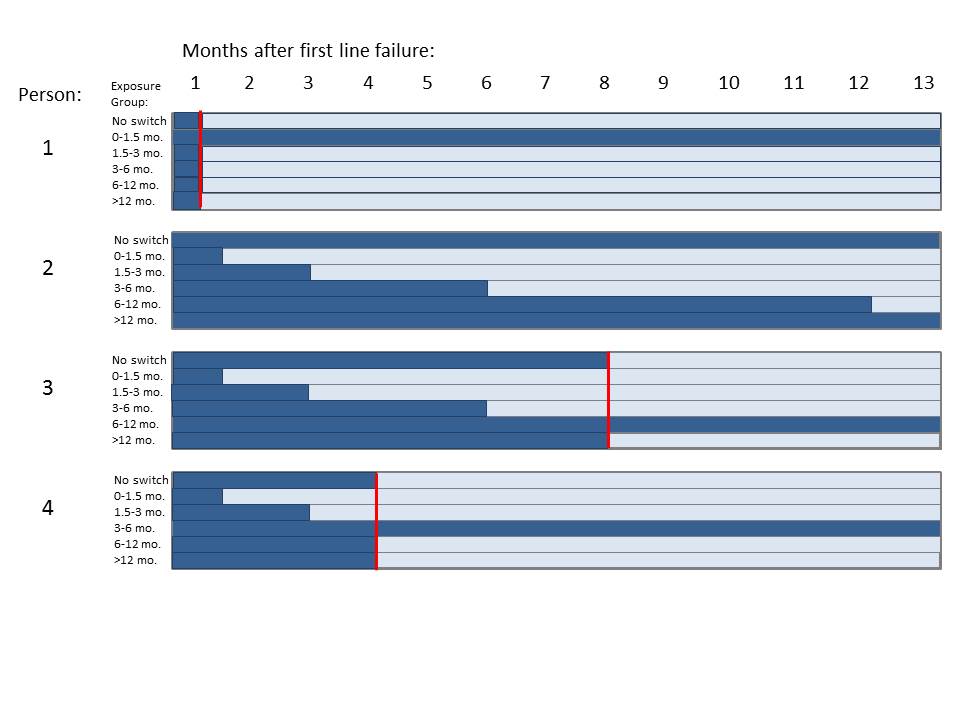

Supplement: S1 Fig — Hypothetical person time contributed to each of the 6 exposure groups in marginal structural models. (DOCX) [file pone.0161469.s001.docx]
