# Supplementary material for: Marginal Structural Models to Assess Delays in Second-Line HIV Treatment Initiation in South Africa
Source: PLoS One. 2016 Aug 22;11(8):e0161469. doi: 10.1371/journal.pone.0161469 (PMC4993510; doi:10.1371/journal.pone.0161469)
Supplement: S1 Table — (DOCX) [file pone.0161469.s002.docx]

| S1 Table. Alternative stratifications for adjusted marginal structural models for hazard ratios of death after first-line failure. | | |
| --- | --- | --- |
| Viral load at first-line ART failure: | ≥ 60 000 copies/mL | < 60 000 copies/mL |
|  | (n = 1513) | (n = 4382) |
| Months to switch | aHR (95% CI) | aHR (95% CI) |
| 0 to 1.5 | Ref | Ref |
| 1.5 to 3 | 1.54 (1.10, 2.14) | 0.80 (0.54, 1.18) |
| 3 to 6 | 1.31 (0.93, 1.83) | 1.05 (0.73, 1.51) |
| 6 to 12 | 1.39 (0.99, 1.95) | 1.07 (0.76, 1.53) |
| >12 | 1.37 (0.98, 1.95) | 1.15 (0.81, 1.62) |
| Never | 1.44 (1.03, 2.01) | 1.16 (0.82, 1.64) |
| Viral suppression (<400 copies/mL) on first-line ART: | No | Yes |
|  | (n = 2923) | (n = 2972) |
| Months to switch | aHR (95% CI) | aHR (95% CI) |
| 0 to 1.5 | Ref | Ref |
| 1.5 to 3 | 1.19 (0.88, 1.60) | 0.94 (0.58, 1.52) |
| 3 to 6 | 1.21 (0.90, 1.63) | 1.01 (0.64, 1.61) |
| 6 to 12 | 1.20 (0.89, 1.61) | 1.18 (0.77, 1.83) |
| >12 | 1.27 (0.95, 1.69) | 1.11 (0.73, 1.70) |
| Never | 1.31 (0.98, 1.76) | 1.15 (0.75, 1.76) |
| *Hazard ratios adjusted for year of failure, sex, age, viral load at first-line failure, CD4 count at first-line failure, missed visits prior to first-line failure. | | |
