# Supplementary material for: Marginal Structural Models to Assess Delays in Second-Line HIV Treatment Initiation in South Africa
Source: PLoS One. 2016 Aug 22;11(8):e0161469. doi: 10.1371/journal.pone.0161469 (PMC4993510; doi:10.1371/journal.pone.0161469)
Supplement: S2 Table — (DOCX) [file pone.0161469.s003.docx]

| S2 Table. Adjusted marginal structural model hazard ratios for death after first-line failure, limiting to patients with 2 weeks to <8 months between failing viral loads on first-line (n = 4908). | | | |
| --- | --- | --- | --- |
|  | All patients | Peak CD4 ≤ 100 cells/mm^3^ prior to first-line failure | Peak CD4 > 100 cells/mm3 prior to first-line failure |
| Months to switch | aHR (95% CI) | aHR (95% CI) | aHR (95% CI) |
| 0 to 1.5 | Ref | Ref | Ref |
| 1.5 to 3 | 1.10 (0.82, 1.48) | 1.42 (0.89, 2.27) | 0.93 (0.64, 1.37) |
| 3 to 6 | 1.22 (0.92, 1.63) | 1.54 (0.96, 2.46) | 1.07 (0.74, 1.53) |
| 6 to 12 | 1.31 (0.99, 1.74) | 1.68 (1.04, 2.69) | 1.19 (0.84, 1.69) |
| >12 | 1.32 (1.00, 1.74) | 1.74 (1.09, 2.77) | 1.16 (0.82, 1.63) |
| Never | 1.38 (1.04, 1.82) | 1.74 (1.09, 2.78) | 1.23 (0.87, 1.73) |
| *Adjusted for year of failure, sex, age, viral load at first-line failure, CD4 count at first-line failure, missed visits prior to first-line failure. Stratum for peak CD4 count ≤ 100 cells/mm3 prior to first-line failure was not adjusted for CD4 count at first-line failure due to small strata. | | | |
