# Supplementary material for: Marginal Structural Models to Assess Delays in Second-Line HIV Treatment Initiation in South Africa
Source: PLoS One. 2016 Aug 22;11(8):e0161469. doi: 10.1371/journal.pone.0161469 (PMC4993510; doi:10.1371/journal.pone.0161469)
Supplement: S3 Table — (DOCX) [file pone.0161469.s004.docx]

| S3 Table. Adjusted Cox proportional hazards ratios for alternative virologic outcomes on second-line ART, stratified by peak CD4 count prior to first-line failure. | | |
| --- | --- | --- |
|  | Peak CD4 ≤ 100 cells/mm^3^ prior to first-line failure | Peak CD4 > 100 cells/mm3 prior to first-line failure |
|  | (N = 496) | (N = 2939) |
| Months to switch | aHR (95% CI) | aHR (95% CI) |
|  | **Outcome: Confirmed virologic failure after 3 months on second-line** | |
| 0 to 1.5 | Ref. | Ref. |
| 1.5 to 3 | 2.53 (1.19, 5.35) | 0.82 (0.62, 1.08) |
| 3 to 6 | 2.59 (1.17, 5.74) | 1.01 (0.76, 1.33) |
| 6 to 12 | 1.21 (0.48, 3.03) | 0.86 (0.62, 1.19) |
| >12 | 1.22 (0.39, 3.81) | 0.65 (0.45, 0.93) |
|  | **Outcome: Single elevated viral load (>1000 copies m/L)** | |
| 0 to 1.5 | Ref. | Ref. |
| 1.5 to 3 | 1.12 (0.68, 1.83) | 0.94 (0.77, 1.14) |
| 3 to 6 | 1.25 (0.75, 2.06) | 1.01 (0.82, 1.23) |
| 6 to 12 | 1.24 (0.75, 2.05) | 1.03 (0.83, 1.29) |
| >12 | 1.31 (0.75, 2.30) | 0.80 (0.64, 1.00) |
| *Adjusted for gender, age, viral load level at failure, BMI at failure, year of failure, missed visits before failure, time on first-line ART, clinic | | |
