# Supplementary material for: Marginal Structural Models to Assess Delays in Second-Line HIV Treatment Initiation in South Africa
Source: PLoS One. 2016 Aug 22;11(8):e0161469. doi: 10.1371/journal.pone.0161469 (PMC4993510; doi:10.1371/journal.pone.0161469)
Supplement: S4 Table — Adjusted marginal structural models for hazard ratios of death after first-line failure (a) and adjusted Cox proportional hazards ratios for confirmed failure on second-line ART (b), with weighting by inverse probability of censoring after second-line switch to account for loss to follow-up. (DOCX) [file pone.0161469.s005.docx]

| S4a Table. Adjusted marginal structural models for hazard ratios of death after first-line failure, with weighting by inverse probability of censoring after second-line switch to account for loss to follow-up. | | |
| --- | --- | --- |
|  | Peak CD4 ≤ 100 cells/mm^3^ prior to first-line failure | Peak CD4 > 100 cells/mm3 prior to first-line failure |
| Months to switch | aHR* (95% CI) | aHR (95% CI) |
| 0 to 1.5 | Ref | Ref |
| 1.5 to 3 | 1.25 (0.79, 1.98) | 1.01 (0.74, 1.36) |
| 3 to 6 | 1.28 (0.81, 2.00) | 1.04 (0.77, 1.40) |
| 6 to 12 | 1.37 (0.87, 2.16) | 1.10 (0.82, 1.47) |
| >12 | 1.42 (0.90, 2.24) | 1.08 (0.81, 1.44) |
| Never | 1.43 (0.91, 2.25) | 1.13 (0.85, 1.51) |
| *Adjusted for year of failure, sex, age, viral load at first-line failure, CD4 count at first-line failure, missed visits prior to first-line failure. Stratum for peak CD4 count ≤ 100 cells/mm3 prior to first-line failure was not adjusted for CD4 count at first-line failure due to small strata. | | |
| S4bTable. Adjusted Cox proportional hazards ratios for confirmed failure on second-line ART, with weighting by inverse probability of censoring after second-line switch to account for loss to follow-up. | | |
|  | Peak CD4 ≤ 100 cells/mm^3^ prior to first-line failure | Peak CD4 > 100 cells/mm3 prior to first-line failure |
| Months to switch | aHR* (95% CI) | aHR (95% CI) |
| 0 to 1.5 | Ref | Ref |
| 1.5 to 3 | 2.53 (1.23, 5.18) | 1.08 (0.78, 1.51) |
| 3 to 6 | 1.99 (0.94, 4.22) | 1.11 (0.83, 1.49) |
| 6 to 12 | 1.33 (0.56, 3.14) | 0.85 (0.60, 1.20) |
| >12 | 1.80 (0.64, 5.01) | 0.87 (0.60, 1.26) |
| *Adjusted for sex age, viral load at first-line failure, BMI at first-line failure, proportion of missed visits before first-line failure, time on first-line ART. | | |
